# Maternity protection policies and the enabling environment for breastfeeding in the Philippines: a qualitative study

**DOI:** 10.1186/s13006-023-00594-w

**Published:** 2023-11-10

**Authors:** Cherry C. Maramag, Jyn Allec R. Samaniego, Mary Christine Castro, Paul Zambrano, Tuan T Nguyen, Jennifer Cashin, Janice Datu-Sanguyo, Roger Mathisen, Amy Weissman

**Affiliations:** 1 Nutrition Center of the Philippines, Muntinlupa City, Philippines; 2 Alive & Thrive, Global Nutrition, FHI 360, Manila, Philippines; 3 Alive & Thrive, Global Nutrition, FHI 360, Hanoi, Vietnam; 4 Alive & Thrive, Global Nutrition, FHI 360, Washington, District of Columbia, USA; 5 Asia Pacific Regional Office, FHI 360, Bangkok, Thailand

**Keywords:** Breastfeeding, Lactation support in the workplace, Maternity entitlements, Maternity leave, Maternity protection, Qualitative study, Philippines

## Abstract

**Background:**

The Philippines has enacted maternity protection policies, such as the 105-Day Expanded Maternity Leave Law and the Expanded Breastfeeding Promotion Act of 2009, to protect, promote, and support breastfeeding. This study aimed to review the content and implementation of maternity protection policies in the Philippines and assess their role in enabling recommended breastfeeding practices. It also identified bottlenecks to successful implementation from the perspectives of mothers and their partners, employers, and authorities from the government and non-government organizations involved in developing, implementing, monitoring, and enforcing maternity protection policies.

**Methods:**

This study employed a desk review of policies, guidelines, and related documents on maternity protection, and in-depth interviews. Of the 87 in-depth interviews, there were 12 employed pregnant women, 29 mothers of infants, 15 partners of the mothers, 12 employers and 19 key informants from the government and non-government organizations. Respondents for the in-depth interviews were from the Greater Manila Area and were recruited using purposive snowball sampling. Data were collected from December 2020 to April 2021.

**Results:**

The study shows that maternity protection policies in the Philippines are mostly aligned with the maternity protection standards set by the International Labour Organization. However, their role in improving breastfeeding practices is limited because: (1) not all working women have access to maternity protection entitlements; (2) the duration of maternity leave entitlements is inconsistent with the World Health Organization’s recommended duration of exclusive breastfeeding; (3) there are gaps in policy implementation including: a lack of monitoring systems to measure the availability, functionality, and usage of lactation spaces; limited workplace support for breastfeeding; poor communication of maternity and paternity entitlements; and limited breastfeeding advocacy and promotion; and (4) there is limited integration between maternity protection and breastfeeding promotion interventions.

**Conclusions:**

There is a need to (1) strengthen communication about and promotion of maternity and paternity entitlements for mothers, fathers and employers, (2) improve monitoring and enforcement mechanisms to ensure utilization of entitlements among mothers, (3) develop modalities to extend the coverage of maternity entitlements to the informal sector, (4) fully cover paid leave entitlements from social insurance or public funding sources in line with International Labour Organization recommendations, and (5) revisit the limitations on the coverage of paternity entitlement.

**Supplementary Information:**

The online version contains supplementary material available at 10.1186/s13006-023-00594-w.

## Background

Despite established evidence of the protections that breastfeeding provides to the mother and child [1], the global prevalence of recommended breastfeeding practices remains low. Only 48% of children younger than six months globally are breastfed exclusively [2] and only 6 of 136 recently assessed countries have achieved exclusive breastfeeding rates above 70% [3]. In the Philippines, only 60.1% of infants below six months of age are exclusively breastfed and only 41.8% are continuing to breastfeed at age two [4]. A mother’s return to work is a major barrier to exclusive and continued breastfeeding [5].

According to a report published by the International Labour Organization (ILO) [6], 120 countries of the 185 they assessed provide at least 14 weeks of maternity leave, the prescribed minimum maternity leave duration set in ILO Maternity Protection Convention No. 183 (Convention No. 183). Among these, 52 countries provide at least 18 weeks of maternity leave [6], as recommended in Maternity Protection Recommendation No. 191 (Recommendation No. 191). Most countries provide the required maternity leave cash payment of at least two-thirds of the women’s previous earnings, and more than two-thirds of potential mothers across the world live in countries that finance entitlements through social protection schemes [6], thus reducing employers’ liability. However, access to adequate maternity leave is still far from universal. About 649 million women globally live in countries that do not meet at least one standard on maternity leave set in Convention No. 183 [6]. Maternity leave entitlements also remain inaccessible to most female workers in the informal sector [6–8].

Longer duration of paid maternity leave is associated with reduced infant mortality, improved physical and mental health among mothers, and improved breastfeeding practices [9–13]. In low- and middle-income countries, a one-month increase in maternity leave is associated with a 7.4% increase in early initiation of breastfeeding, a 5.9% increase in exclusive breastfeeding, and a 2.2-month increase in breastfeeding duration [9]. Studies in high-income countries have also shown a positive association between maternity leave duration and breastfeeding practices [14, 15]. In the Philippines, the issuance of the 105-Day Expanded Maternity Leave Law (EMLL) in 2019 increased the duration of paid maternity leave from 60 to 78 days to 105 days, with an additional 15 days for solo parents [16]. The EMLL likewise provided entitlements for maternity cash benefits through combined funding from social security funds and employers [16].

Maternity leave, along with paternity and other parental leave, is also a form of care leave that allows parents to take care of their children without having to relinquish employment [6]. Currently, the Philippines does not have a statutory childcare service system in place for children less than 5 years of age [6].

The provision of time, space, and support for breastfeeding for working mothers when they return to work is associated with improved breastfeeding practices [17–19]. The Philippines’ 2009 Expanded Breastfeeding Promotion Act mandated the establishment of lactation rooms and paid lactation breaks in workplaces [20]. However, a 2015 Philippine national survey showed that only 28.9% of working mothers were aware of workplace lactation rooms [21]. A study also showed that in 2015, only 10.7% of working mothers had ever used lactation rooms and only 35.1% of currently breastfeeding mothers used lactation breaks [22]. In 2015, the Department of Labor and Employment (DOLE) issued a policy to guide the establishment of lactation rooms in workplace settings [23] and has included these spaces in monitoring labor law compliance of private enterprises.

Legislation on maternity protection and workplace lactation support in the country exists, but there has been no study conducted on the effects of these policies on breastfeeding practices of working mothers. This study was conducted to review the content and implementation of maternity protection policies in the Philippines and assess their role in enabling recommended breastfeeding practices, including their potential impact on improving breastfeeding support for working women. The study also aimed to identify bottlenecks to successful implementation from the perspectives of mothers and their partners, and employers, as well as government and non-government organization authorities involved in the development, implementation, monitoring, and enforcement of maternity protection policies.

## Methods

### Study design

The data collection, analysis and interpretation were guided by a conceptual model for this study published in a research protocol by Nguyen et al. [24]. The conceptual model included the analysis of maternity protection policies and the structural supportive factors required to improve breastfeeding practices and the identification of facilitating factors and barriers to achieving this desired improvement. Data collection was done through a desk review of maternity protection policies and other related documents. In-depth interviews (IDI) of mothers and their partners, employers, and government and non-government organization authorities involved in the development, implementation, monitoring, and enforcement of maternity protection policies were also conducted to identify facilitating factors and barriers to the implementation of maternity protection policies.

### Setting

The Philippines is an archipelagic country located in Southeast Asia with an estimated population of 115.6 million [25]. Classified as a lower middle-income country, its gross national product (GNP) was worth USD 404.3 billion in 2022 [26]. The services, industry and agriculture, forestry, and fisheries sectors are the main drivers of the Philippine economy [27]. Women represent about 44% of the country’s total workforce [28] of 47.7 million as of 2021 [29]. The mortality rate among children under five years of age (under-five mortality rate), infant mortality rate, and neonatal mortality rate were estimated at 26.5, 21.05 and 12.56 per 1000 live births in 2020, respectively [30–32].

Due to restrictions placed to prevent the spread of COVID-19 during the data gathering period, the coverage of the study was limited to study sites within the Greater Manila Area (National Capital Region and the neighboring provinces of Bulacan, Cavite, Laguna and Rizal). These regions were among those with the lowest exclusive breastfeeding rates [22]. The Greater Manila Area is highly urbanized, has a high population density, and includes industrial zones and business districts with workplaces employing women. The specific study sites were selected through purposive sampling with considerations regarding internet connectivity to facilitate data collection. The study sites were not representative of the Philippines as a whole, where about 52% of the total population resides in rural areas [33].

### Desk review of policies

Data were gathered for the desk review from December 2020 - April 2021. Copies of policies, laws, survey reports, and program documents relevant to maternity protection were retrieved from national and local government offices. The effectivity date, legal status, coverage, implementation strategies, and monitoring guidelines were sourced from these documents. The extracted information was compared with the standards set in Convention No. 183 and Recommendation No. 191. Data on the number and proportion of working mothers receiving maternity entitlements and details related to monitoring and reporting were retrieved from agencies or their official websites. In addition, we reviewed information on the implementation, coverage, monitoring, and enforcement of those policies.

### In-depth-interviews

#### Sampling and participants

Purposive snowball sampling was used to select respondents for IDIs. We conducted IDIs with 87 respondents. Eighty-two of the interviews were audio recorded, and 48 were face-to-face interviews (Table 1). The research team recorded notes for the five interviews that were not audio recorded.

**Table 1:** Number of IDI respondents by type: key informants from the government and non-government organizations, employers, pregnant women, mothers of infants and their partners

Type	Planned	Actual		
		Total	Female	Face-to-Face
Pregnant women	12	12	12	12
Mothers of infants aged 0 to 5 months	12	13	13	11
Mothers of infants aged 6 to 11 months	12	16	16	12
Partners of mothers with infants aged 6 to 11 months	12	15	0	13
Employers	12	12	10	0
Key informants from the government and non-government organizations	12	19	15	0
Total	72	87	66	48

The mode and criteria for selection per type of respondents are enumerated below:

##### Mothers and their partners

This included pregnant women who were at least 18 years of age at the time of the interview; and mothers of infants less than 12 months old who were at least 18 years of age at the time of interview and their partners. They were approached by the staff of the Nutrition Center of the Philippines (NCP) through spot mapping, referral by local health centers, or personal contacts within the communities in the study area (Greater Manila Area). Participant selection considered diversity in employment status (full-time/part-time), work set-up (work from home/online/in person), and current breastfeeding practices (breastfeeding/not breastfeeding/mixed feeding). Working mothers were interviewed away from their workplaces (either at home or a neutral community location) to reduce bias in responses.

##### Employers

The interviewed employers were identified through referral by regional program managers of health, national employers’ associations, and personal contacts of NCP staff and were interviewed outside their workplaces to reduce bias in responses. The employers were either leaders, general managers, or human resources personnel of workplaces with at least 50 female employees located within Greater Manila Area.

##### Key informants from government and non-government organizations

This group of respondents included authorities from the government, representatives of non-government organizations participating in the development, implementation, monitoring, and enforcement of maternity protection policies in the country. The NCP, in consultation with Alive & Thrive, prepared the list of key informants which included representatives from the Philippine Senate, Department of Health (DOH), DOLE, Civil Service Commission (CSC), UNICEF, World Health Organization (WHO), ILO, Social Security System (SSS), and other relevant organizations.

Respondents were contacted either through email, telephone, or face-to-face. All refusals were replaced with other candidates until the minimum number of respondents per type of respondent was met. The language used in the majority of the interviews was Filipino.

### Data collection

Before the interview was scheduled, each participant was oriented on the purpose and procedure for the IDI, including the confidentiality of the collected information and the use of anonymized information for the study. Respondents were provided with an informed consent form either in-person or via email. The signed informed consent forms were collected prior to the conduct of the interviews. Six interviewers hired by the research team, who happened to be all female, conducted the IDIs. They were all experienced in data collection including in the conduct of IDIs and have undergone specific training before the conduct of the IDIs for this research. All interviews were one-on-one interviews. Each interview took around an hour to finish and was done either online or onsite, according to the respondents’ preference. Verbal consent for the interview and audio recording was audio-recorded at the time of the interview. Participants who consented to the interview were assigned a personal identification number (PIN), which was linked to the personal data collected from each participant. These data are password protected and are only accessible to the research team. Interviews were conducted between 3 December 2020 and 17 March 2021. IDI respondents were given cash tokens worth 3.7 USD for their participation. Table 2 presents the type of information elicited from the IDIs per type of respondent. The data collection tools used for the IDIs are included as Additional File 1.

**Table 2:** Domains of in-depth interviews per type of respondent

Domains	Pregnant women and mothers of children aged 0–11 months	Partners of mothers	Employers	Key informants from government and non-government organizations
Policy(ies) development				√
Policy(ies) implementation			√	√
Policy(ies) monitoring and enforcement			√	√
Perceptions of and experience with policy(ies)	√	√	√	√
Suggestions for improvement	√	√	√	√
Sharing responsibilities in caring for children and domestic tasks	√	√		

### Data analysis

Audio recordings were uploaded to an internet-based storage drive accessible only by authorized team members and transcribed in the language used during the interview. Full transcripts were also translated into English, as needed. Nvivo software (v. 1.4.1, QSR International, 2021) was used to analyze the transcriptions. Line-by-line initial coding of each transcript was done until theoretical saturation was monitored, and the emerging themes were identified. Each transcript was coded independently by two coders. The identified themes from the two coders were compared and discussed by the team and reviewed against data collected from initial key findings of the team and available documents to produce more selective conceptual codes to explain larger segments of data. The results of the IDIs were then compared with the findings from the desk review to identify common themes and related findings.

## Results

Out of 87 in-depth interviews, 66 respondents were female (Table 1). The mothers, along with their partners, were equally distributed among the target characteristics as enumerated in Table 3. Among 12 interviewed employers, six were from manufacturing or merchandising workplaces, two were from business process outsourcing companies, and four were service-related employers. Among the 19 interviewed key informants, three were government legislators, ten were representatives from government agencies, and six were from non-government organizations.

**Table 3:** Characteristics of interviewed pregnant women, mothers of infants and their husbands/partners participating in in-depth interviews

	Number of women	Number of partners/husbands
Pregnant woman		
Employed full-time:		
Production area	2	
Office	2	
Business process outsourcing	2	
Work from home	2	
Work outside the home, first pregnancy	2	
Self-employed and work from home	2	
Mother with child aged 0 to < 12 months		
Employed full time:		
Attended IYCF counseling during pregnancy	3	2
Business process outsourcing, and worked night shift during pregnancy	3	1
Breastfeeding, work outside	3	1
Breastfeeding, work from home	3	1
Breastfed during maternity leave, shifted to formula after return to work, work outside the home	3	1
Returned to work before completing maternity leave, work outside	2	1
Work outside the home, not breastfeeding	3	1
Self-employed:		
Work from home, currently breastfeeding	3	2
Work outside the home, breastfeeding	3	2
Work outside the home, not breastfeeding	3	2

Study results are presented by theme, based on the combined analysis of the findings of the desk review and the IDIs.

### Maternity protection entitlements have improved to align more closely with international standards

Maternity protection policies in the Philippines are aligned with most of the standards set in ILO Convention 183 (Additional files 2 and 3). The issuance of the EMLL in 2019 increased paid maternity leave duration to 105 days from the previous 60 days for vaginal birth and 78 days for cesarean birth, bringing the legislation into alignment with ILO Convention No. 183, but not ILO Recommendation No. 191. The EMLL also provided an option of additional 30 days unpaid leave for all recipients and an additional 15 days of paid maternity leave for solo parents and allowed up to 7 days of leave credits from the mother’s 105 days maternity leave to be transferred to the child’s father (or an alternate caregiver in the case of absence or incapacity of the father), on top of the 7 days paternity leave credits for fathers. The Paternity Leave Act of 1996 granted 7-day fully paid paternity leave to all male married employees in private and public sectors for the first four births of their legitimate spouse [34].

The EMLL mandated mixed-source funding from social security and employers starting in 2019. ILO recommends that financing for paid maternity leave come from public or social security funds. However, the Philippines’ law required employers from the private sector to shoulder the salary differential if the mother’s salary credit from her contributions to the SSS fund is insufficient to cover 100% of her salary during maternity leave. The SSS is a government-run social insurance program in the Philippines catering to workers in the private sector and the informal sector.

The Labor Code of the Philippines states that acts of discrimination against women, such as receiving reduced compensation, are considered criminal acts [35]. EMLL also prohibits discrimination in employing women to avoid the provision of maternity entitlements.

Medical entitlements for mothers are also provided through the Philippine Health Insurance Corporation (PhilHealth) [36], the government-managed social health insurance program. Mothers who are active paying members of PhilHealth, regardless of SSS membership, are entitled to receive the following services free of charge at PhilHealth-accredited government facilities: prenatal and postnatal care, childbirth, and hospitalization. Hospital expenses related to childbirth in an accredited private facility are subsidized by PhilHealth. Mothers who are not PhilHealth members or have no qualifying contributions may be registered as sponsored members under point of care enrollment in government facilities. Those who do not qualify will need to pay an amount equivalent to one year premium or the total premium covering the missed and unpaid quarter/s of the calendar year to utilize the medical entitlements.

**Fig. 1:**
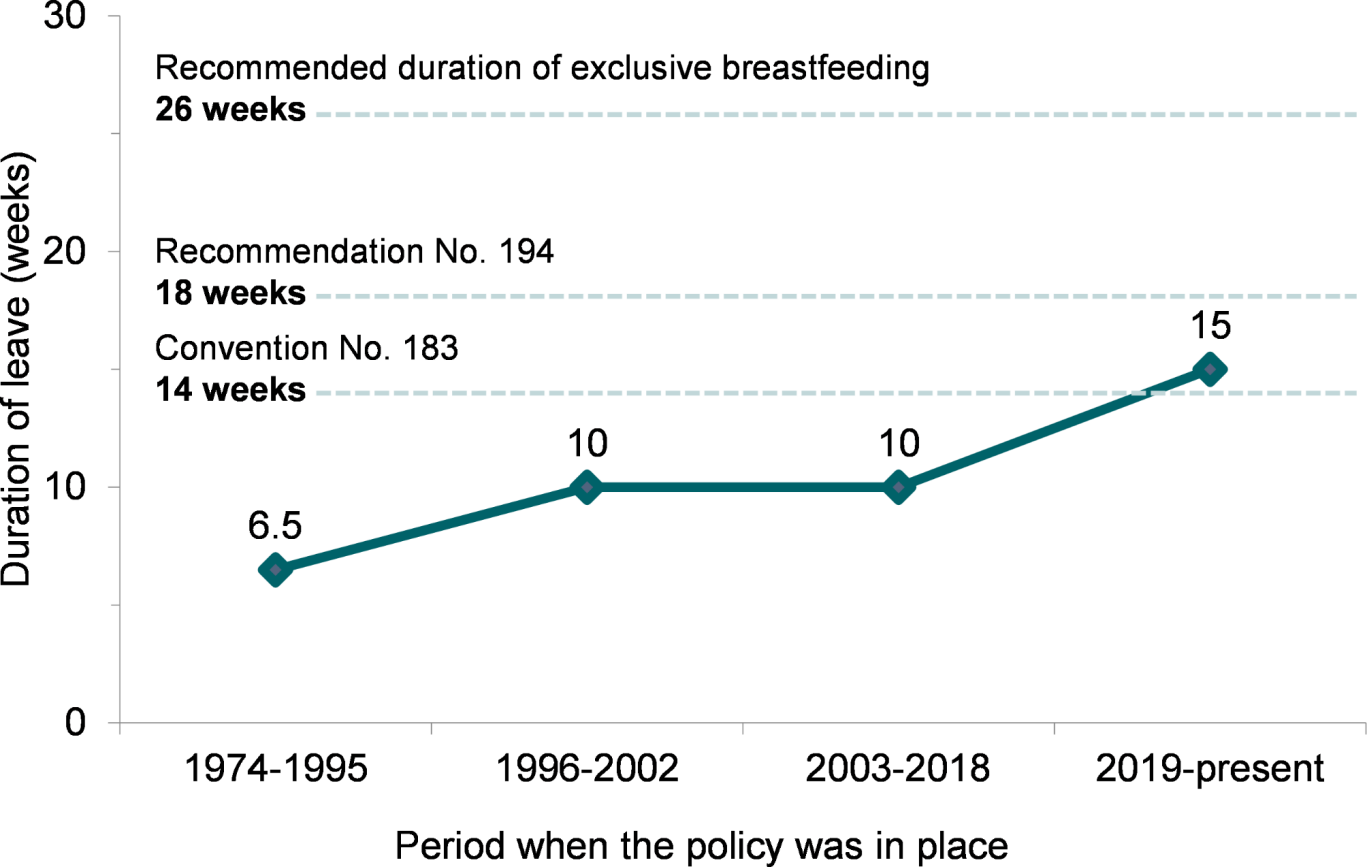
History of paid maternity leave provision in the Philippines 1974–2022 in relation to international recommendations

A timeline of the enacted maternity protection provisions is presented in Fig. 1 and Additional file 4.

### Not all working women can access maternity protection entitlements

Despite recent policy improvements, the ILO’s recommendation that maternity entitlements must cover the majority of working women has not been met. Maternity leave cash entitlements for private and informal sector workers were only granted to mothers enrolled in SSS with at least three-monthly contributions paid in the 12-month period preceding the semester of childbirth [16]. Membership in SSS is also not universal. There were 24 million privately employed and 12.8 million self-employed Filipino workers in 2020 [37]. SSS estimates that there are only 9.6 million privately employed and about 2.4 million self-employed and voluntary paying members as of May 2021 [38]. Among the female working age population, SSS coverage was estimated to be only 54% as of 2020 (SSS, personal communication). Female workers in the government sector, who were entitled to the same fully paid maternity leave, represented only 3% of the female working age population (CSC, personal communication).

Mothers under contract employment, such as independent contractors or freelance employees whose SSS contributions were not updated, were not eligible to receive maternity entitlements. Some respondents reported negotiating maternity leave duration that is less than the mandated 105 days and not receiving any cash payments during leave.

*I am just new here, under Job Order, so I don’t have these. They only provided me 3 months [of maternity leave], but it does not have maternity pay. So, I didn’t have a salary [while on maternity leave]. (Mother, PIN 5028)*

Interviews with female employees also revealed that some employers used short-term contracts to avoid provision of maternity entitlements to employees.

*From what I heard in the factory, they don’t pay contributions for the benefits. Because I know in the factory, contracts end every 5–6 months, after which they will have to be renewed again. (Pregnant woman, PIN 4011)*

Most respondents were not aware of how self-employed workers, such as street vendors and farmers, could access maternity entitlements. There was limited awareness of the SSS mechanism among interviewed self-employed parents. Those who had self-enrolled in SSS faced difficulty maintaining the monthly contributions.

*I think it will only interfere to our daily needs if we will have to pay [SSS contributions]. (Mother, PIN 5012)*

Most IDI respondents from the informal sector were more aware of and utilized PhilHealth medical entitlements for childbirth compared to maternity leave entitlements from social security programs.

### Uptake of paternity leave entitlements are limited by marriage requirements and informal employment

Most fathers were not aware of their right to paternity leave and related entitlements. Among those who were aware, respondents mentioned barriers to accessing entitlements, including not being legally married, as required by the Paternity Leave Act; or working in the informal sector. One respondent also mentioned the type of employment of the mother as the reason for his ineligibility to receive paternity leave entitlements, a violation of the law since there is no such requirement.

*In our delivery service company, we don’t have something like that [paternity leave entitlement], we were just like business partners with them, but we were not employed under them. (Father, PIN 6009)*

*I asked the HR (human resources) before, she said I can only avail paternity leave when my wife receives maternity leave. (Father, PIN 6010)*

### Despite awareness of the value of maternity protection, employers perceive disadvantages to policies

Employers, mothers, and fathers mentioned the following advantages of maternity entitlements: longer bonding time with their infants; increased time for mother to recuperate; and financial support. Only a few specifically mentioned increased time for breastfeeding as one of the benefits. Employers also mentioned benefits of maternity protection policies including increased employee morale; improved maternal and child health; good publicity to recruit and keep talented workers; and removal of the burden of choice between career or family.

*It positively impacts the bonding between the mother and child. . we have what we called milestones that any mother would not want to miss with their child. (Employer, PIN 3007)*

*It will also help psychologically, since you have a sense of security. Mothers usually experience postpartum [depression] at this time. And it will help if they have period to rest. (Employer, PIN 3011)*

*By receiving these kind of [maternity] benefits, the employee would feel valued and cared by their company. I think they will give it back and do their work better as an appreciation to their company. (Employer, PIN 3012)*

Some employers felt that having female employees go on maternity leave disrupts workplace operations and acknowledged the possibility of discrimination against women during hiring. Employers also perceived that non-parent workers who may receive additional workload while mothers are on maternity leave may feel upset, as they are unable to avail of such entitlements.

*In my opinion, I think the non-parents will be upset since they are not entitled [to receive] these benefits. Also, there will be an added cost, aside from their paid leave. For the production operations, we still have to allot time for retraining and transition to work. This also includes planning and designing the work arrangement. (Employer, PIN 3019)*

*Actually, in a profit-oriented atmosphere, like in my workplace before, they request their temporary workers who were pregnant women to resign, since they will have to pay them for three months, and it is a liability. (Employer, PIN 3009)*

### There are policies in place that mandate workplace breastfeeding support

The Expanded Breastfeeding Promotion Act of 2009, which mandates the establishment of lactation rooms and implementation of lactation breaks in workplaces, is aligned with ILO recommendations. This policy requires that workplaces create a breastfeeding policy and prohibits marketing of breastmilk substitutes. Compliance with the Act is required to issue or renew business permits. Private and public workplaces may apply for a renewable exemption from establishing lactation rooms to DOLE and CSC, respectively [39, 40]. The criteria for exemption are different between the two agencies. DOLE, through Department Order No. 143, 2015, grants exemptions based on the presence of lactating and pregnant employees and presence of female guests in the premises during operating hours [39]. CSC, through the issuance of Memorandum Circular No. 12 series of 2015, considers the physical size of the agency, number of female employees, and average number of women visiting the workplaces [40].

Workplaces may apply for Mother-Baby Friendly Workplaces Certification, which are valid for two years, by complying with the requirements of the Expanded Breastfeeding Promotion Act of 2009 and fulfilling additional requirements set by the DOH; requirements include the provision of a manually operated breast pump, paper towels, and a covered trash can in the lactation rooms [41]. Review and assessment of applications is assigned to local government units (LGUs), while the onsite inspection and approval of certification is conducted by DOH Centers for Health Development (CHD). As an incentive, the cost incurred to comply with the Act would be deducted from taxable expenses. However, this was only effective within 6 months after approval of the law’s implementing rules and regulations (IRR) in 2011 [42]. There is no available information on workplaces that availed this tax incentive.

### Implementation of workplace lactation support policies varies according to workplace and type of work

Some employers mentioned the presence of lactation rooms and refrigerators in their workplaces, while others referred breastfeeding mothers to clinics or unused or vacant rooms for breastfeeding and breastmilk expression.

*Even if we don’t have a lactation room, we always make sure that they have a private room to do this. Usually, we have “lactation” signage. From our previous lactating mother, sometimes she doesn’t have time to lactate. So usually, she brings this signage and looks for a vacant room, and she will just post this signage. (Employer, Female, PIN 3006)*

Based on enacted policies, local government units were encouraged to set up lactation spaces and programs for informal workplaces in partnership with the private sector. However, this is not widely implemented across all LGUs.

*It will depend on the ordinance and the cooperation of the local government. Because these informal sector workers have no formal employers to talk to when they do not have these facilities [lactation spaces] for workers. (Programmer, Female, PIN 1021)*

Employers have policies in place to complement national regulations. Some provide flexible working arrangements, free transportation services, additional cash assistance, free medical check-ups under the workplace’s health insurance package, and free childcare benefits.

*Aside from maternity leave benefits, the company also gives childbirth assistance. I think it is PHP 1,000 to 2,000 (USD 20 to 40). (Employer, Female, PIN 3023)*

Some workplace settings made breastmilk expression difficult due to workload and work schedule, with either nonexistent or inadequate break time. Output-based jobs with flexible working hours in the formal and informal sector also forces mothers to choose between earning more income and breastfeeding.

*I think in this situation, cashiers have no break time unless their duty is finished. (Employer, Female, PIN 3010)*

*In that kind of work [production area], I think it is difficult to let down milk, because the mother does not have ample time to rest. (Employer, Male, PIN 3009)*

*Since the wages depend on product outputs, there is a possibility that the mother will opt for formula milk because of the hassle of extracting milk. (Employer, Female, PIN 3007)*

Respondents also mentioned that maternity leave is not applicable for workers in the informal sector, making informal workers’ financial needs a contributing factor in their decisions to return to work early. Several informal work settings also had no suitable place for breastmilk expression and storage or were inappropriate environments for children.

Additional costs incurred by employers to establish and maintain lactation rooms, and to provide the cost differential from SSS claims was cited as a disadvantage, especially for small and medium enterprises. The mandatory establishment of lactation spaces was considered impractical by some key informants from the government and non-government organizations and employers due to the required investment and space limitations within some types of businesses. The small number of mothers who utilize the facility was also perceived as minimizing the impact of the investment.

*Most of the employees here are already past their reproductive age, you will just waste that room which could be used for other purposes…. For example, the clinic is located on the first floor, but the lactating mother is located on the fourth floor. Of course, she will put the expressed milk in the refrigerator on the fourth floor. Let’s say in that quarter, she was the only lactating mother in the workplace. It is not wise to establish a lactation station on the fourth floor just for her (Local level policy maker, Female, PIN 1023)*.

### Some employers do not support lactation at work

Some employers expressed unsupportive views toward breastfeeding and breastmilk expression at work. Employers and mothers recognized the difficulty in continuing breastfeeding once a mother returns to work, implying a lack of awareness of legal entitlements among mothers and a lack of knowledge and support for breastfeeding on the part of employers.

*When a mother returns to work, they already perceive that breastfeeding is difficult in their work in the production area. They have to bring necessary materials such as containers and breast pumps which is inconvenient. They lack awareness on what would be the best for the baby. (Employer, Female, PIN 3028)*

Some employers relied on the working mothers’ initiative and the accessibility of information from the internet rather than actively promoting and disseminating information related to maternity protection and workplace lactation support. One respondent equated maternity entitlements with merely compensation or receiving monetary support and did not seem to recognize that mothers need other workplace support during the maternity period.

*There is no need for a very laborious information campaign, because as I’ve said this information is already available on the internet. So, we just tell them to sign the maternity benefits form. This is what is going to happen, this is the process, the date that you will be able to get it and all of that. Because what they expect really is when will they get the maternity benefits. (Employer, Female, PIN 3011)*

### Many mothers cannot balance between breastfeeding and work

There was a sense of acceptance among mothers that breastfeeding will stop once they return to work. Mothers working in a variety of different occupations mentioned the difficulty and inconvenience of expressing and storing breastmilk during work. Among women who took maternity leave, some breastfed only until the end of their leave.

*Since I am working, I have no choice but to switch to infant formula. (Mother, bank teller, PIN 5004)*

*I am already practicing my child for infant formula, thus I already opted for mixed feeding. Because, after 3 months, I will return to my work. (Mother, supermarket employee, PIN 5003)*

*For mothers who need to work, it is usually difficult to pump and pump, then you will have to carry it, also there are uncontrolled times when you can express breastmilk. (Mother, part-time worker in government office, PIN 5007)*

### Systematic inter-agency enforcement and monitoring system not in place

Both SSS and PhilHealth record the number of maternity entitlement claims paid per year. DOLE has integrated the monitoring of maternity protection policy implementation, including monitoring of workplace lactation support, within its annual inspection for monitoring of workplaces in the private sector. DOLE recorded a high percentage (85 to 99%) of compliance among workplaces inspected from 2014 to 2020 [43]. However, less than 10% [43] of the estimated 1,500,506 private sector workplaces in the country [44] were inspected. Reasons for this low coverage include the insufficient number of inspectors to cover the high number of private sector workplaces and the reportedly lengthy labor inspection checklist requiring inputs from different types of professionals, possibly resulting in lower quality inspection. For the public sector, there is currently no established system for monitoring compliance.

The Department of the Interior and Local Government (DILG) issued a memorandum to guide the establishment of workplace lactation programs in local government offices, inclusion of workplace lactation programs for business registration, and private-public partnership in setting up and sustaining lactation management programs for the informal sector [45]. However, based on the result of this study, there is no established system to monitor implementation. Furthermore, the issuance does not mandate partnership of workplaces with local health offices for technical assistance on breastfeeding programs and development of educational materials.

There are no guidelines to monitor the implementation of and compliance with maternity protection policies. The roles and responsibilities of different government agencies have not been clarified. These indicate that there are gaps in understanding on how these agencies should contribute to monitoring and enforcement.

*I am not aware of any monitoring guidelines developed for all levels as contemplated under Sect. 30, Rule VIII of the IRR. (Programmer, Female, PIN 1021)*

While existing policies include provisions for the establishment of mechanisms for monitoring service utilization, the monitoring systems currently in place are limited in their capability to measure functionality and uptake of maternity protection entitlements.

Regional DOH-CHDs conducted stringent inspections but only among workplaces that applied for the Mother-Baby Friendly Workplaces Certification, which is voluntary. The setting up of inter-agency monitoring teams for all levels as mandated by law had not been done. There were recommendations for the integration of monitoring and enforcement activities in the mechanisms for monitoring other current programs of the DOLE and DOH.

### Key informants from the government and non-government organizations agree on the need to increase breastfeeding promotion and support in workplaces

Policy makers mentioned the need for increased breastfeeding promotion activities among employers, fathers, and mothers to encourage breastfeeding and create more supportive work environments that encourage mothers to utilize their maternity entitlements. At present, employers comply with the mandate because it was included in the DOLE checklist but may not fully recognize the purpose and benefits of the policy. They also had insufficient knowledge to provide proper assistance to breastfeeding employees. One employer provided a care package that included diapers and breastmilk substitutes.

*On top of their maternity benefits, we have a care package that was given for the babies first 6 months. So, there was a budget for milk and diapers that we provide for them. (Employer, Female, PIN 3011)*

*We conducted a survey, they have provided space for the breastfeeding station, but no advocacy activities are being conducted on the benefits of breastfeeding and how it will be done. The HR [human resource] personnel do not know how long the storage is and how the milk will be transported if they are far from work. We need to invest in teaching mothers how to do it [breastmilk expression and storage]. Do they need a cooler when transporting it? They want to breastfeed, but they do not know how to do it. (Civil society advocate, Female, PIN 1028)*

*We can contribute by ensuring that there are facilities in the workplace, but it is the personal decision of the mother, whether or not she will breastfeed because it is such a long process. I believe that people should be re-educated on the benefits of breastfeeding. (Programmer, Female, PIN 1021)*

*We need to have a supportive environment, not just the facility. They must view it in a positive way, as a way of life. We need to have that perspective and an accepting organization, wherein you can freely take a break because of your lactation duty. (Local level policy maker, Female, PIN 1023)*

## Discussion

Findings from our study indicate that maternity protection policies in the Philippines are aligned with most of the standards set in ILO Convention No. 183. These policies should be expected to mitigate the impact of return to work as a major structural barrier to breastfeeding [1]. However, the country’s maternity leave duration is not aligned with the recommended minimum leave duration of 18 weeks in ILO Recommendation No. 191. It is also inconsistent with the WHO recommendation that infants should be exclusively breastfed for the first 6 months, which requires proximity of mother and infant. Increasing the maternity leave duration to the appropriate recommendation will promote improved breastfeeding practices and increase exclusive breastfeeding rates [9, 46].

Our findings illustrate how gaps in implementation and unsupportive social norms limit the impact of maternity protection policies on breastfeeding practices. These include a lack of monitoring systems to measure the availability, functionality, and usage of lactation spaces; barriers to the uptake of maternity protection entitlements among workers in the informal sector and short-term/contract workers in the formal sector; lack of workplace support for breastfeeding; poor communication of maternity rights; and limited breastfeeding advocacy and promotion. The lack of integration with breastfeeding promotion interventions likewise limits the role of current maternity protection policies in improving breastfeeding practices.

Paternity leave enables fathers to be with their partners during the period after childbirth, and their presence and support during this period considerably impacts the mother’s decision to breastfeed [47]. Our findings indicate the need to increase fathers’ awareness of paternity leave entitlements. The policy aspect requiring legal marriage to be able to utilize paternity leave entitlements are neither child-centered nor aligned with the principle of non-discrimination [48] to ensure the right to equal treatment of children born out of marriage.

Mothers in our study seemed to have accepted that they need to use breastmilk substitutes or even stop breastfeeding their infants when they return to work. Workplace settings with high workloads, inflexible break times, lack of dedicated time for breastfeeding breaks, and absence of lactation spaces discouraged breastfeeding and breastmilk expression [19]. Mothers in output-based work also prioritized work performance, and pay or career advancement, over breastfeeding. These findings indicate that additional policy action is required to improve implementation and increase coverage for women in all forms of employment.

Generally, employers were compliant with maternity protection policies, as these were required for their business permits and licenses. However, beyond compliance, there were employers who were not cognizant of their role in providing other forms of breastfeeding support in their workplaces, and their female employees’ right to this support. Lactation facilities were merely seen as spaces for breastmilk expression, and not as spaces for mothers to be safe and comfortable and as investments for children’s health. Our findings are consistent with an earlier study showing that insufficient knowledge of employers on breastfeeding prevented the translation of maternity entitlements to improved breastfeeding practices [17].

The need for technical assistance to support lactation in the workplace and breastfeeding advocacy among employers to improve the uptake of breastfeeding breaks and spaces was recognized by study participants. There is a need to create organizational policies to (1) improve communication and dissemination of information on available interventions, (2) facilitate delivery of practical breastfeeding education among mothers, including the risks of formula feeding and guidance on breastmilk expression and storage; (3) orient and sensitize managers and leaders on the importance of workplace lactation support; and (4) create a supportive environment that enables women to continue breastfeeding when they return to work [13, 19, 49].

Partnerships among DOH, related organizations, and workplaces should be required to ensure continuity of advocacy for breastfeeding programs. Mandating workplaces to set up lactation rooms without providing technical assistance on operationalization, education, and tools to promote breastfeeding in the workplaces is a missed opportunity.

The provision of tax incentives in the Expanded Breastfeeding Promotion Act of 2009 could have been a good strategy to encourage early compliance of workplaces to the provisions of the law, but based on the results of the desk review, there were no records on the number of workplaces which made use of the incentive. This may indicate that not many workplaces applied for the incentive. An extension of this tax incentive or development of new incentive program may be explored to promote and sustain functionality of services or provisions on breastfeeding support, protection, and promotion in workplaces. The program should be promoted and communicated with well-defined guidelines on how to avail of the incentives to encourage workplaces to continue and improve workplace breastfeeding support.

Policymakers, employers, and mothers recognized that the lack of breastfeeding advocacy and promotion results in negative attitudes towards policies and insufficient support to both employers and workers. There was a high recognition of the benefits of maternity policies among employers. However, the burden of financing part of the paid maternity leave ascribed to employers may inadvertently lead to discrimination against female workers, despite the policy on non-discrimination. This emphasizes the importance of utilizing social insurance or public funds to fully finance maternity leave entitlements, which is currently not the case for the private sector. Hiring and training of workers who will temporarily take on the work assignments of the mothers on leave also entail additional cost. An employer may resort to assigning the duties and responsibilities of the mother to existing employees instead of hiring a temporary worker to save cost, creating negative perceptions about employees using maternity leave entitlements.

Our findings also revealed mothers’ perceptions that breastfeeding is difficult and inconvenient. This resulted in underutilization of lactation facilities which the workplaces have invested in. Even paid maternity leave was not associated as an opportunity to practice breastfeeding, but as another form of compensation.

There was limited communication on breastfeeding support among employees despite the presence of breastfeeding policies. According to Anderson et al., mothers were unsure how the support would be enacted in individual situations and the interpersonal communication about breastfeeding support was difficult to initiate and maintain [50]. Awareness and appreciation on the importance of breastfeeding must be instilled at all levels to facilitate improved implementation of maternity protection policies and related workplace entitlements. There is a need to change individual, interpersonal, and organizational perspectives to achieve equitable working conditions for breastfeeding [50]. Employers should be oriented on maternity protection policies and their benefits for individuals, businesses, and society, and supported to utilize their workplaces as a venue for breastfeeding advocacy among workers. Interpersonal communication between employers and employees could enhance breastfeeding support [51]. A breastfeeding policy in the workplace that is well-communicated to all employees would also promote a positive attitude towards breastfeeding [52].

Allowing individuals to enroll in SSS and PhilHealth as voluntary members widened the population who can avail of health protection from social security programs, regardless of employment status. However, the premium contributions required for SSS and PhilHealth coverage make them inaccessible to most women working in the informal economy. Respondents reported limited awareness of social security programs and financial difficulties as primary reasons for not enrolling or maintaining membership in social security schemes. The financial costs, the scheme-related awareness, understanding and trust, and the need and benefit from the insurance system were factors that influence enrollment and retention in social security and health insurance schemes among informal workers [53, 54]. The improvement in the PhilHealth benefit packages increased the number of maternity claims. However, more than 40% of workers in the informal sector were found to have no health insurance coverage [55]. Further, a 2015 survey in the Philippines showed that only 12% of self-employed mothers received maternity leave entitlements while about 25% of working mothers in the public sector and 45% of working mothers in the private sector with children aged 0 to 36 months did not benefit from paid maternity leave [23].

There was also limited accountability and support on the establishment of lactation spaces for the informal sector as it is strongly reliant on local government’s priorities. There might be a need to revisit the policies to address this gap and develop a different operational modality for provision of maternity entitlements specific to the informal sector. A cost estimate study for the Philippines showed an investment on non-contributory 26-week maternity cash transfer for the informal sector will only cost less than 0.1% of the country’s gross domestic product, considerably lower than the share of cost of not breastfeeding which is 0.7% [8]. In May 2021, a bill was filed to provide cash aid, an equivalent minimum wage for 22 days, to pregnant workers from the informal sector who are not SSS members [56]. A house bill for the Magna Carta of Workers in the Informal Economy was also filed in order to transition informal workers to the formal economy [57].

We acknowledge several limitations to our study, including the purposive sampling design and limited area coverage which may affect the generalizability of the data. The study sites in the Greater Manila Area are not representative of the Philippines as a whole, which remains predominantly agricultural outside of major urban areas. However, given that national program managers in government and non-government agencies were included in the study, they might have provided context on the general situation in the Philippines.

## Conclusions

Although maternity protection policies were in place and have improved, there is a need to (1) improve communication and promotion of maternity protection rights of mothers, paternity leave entitlements of fathers, and duties of employers, (2) strengthen monitoring and enforcement mechanisms to ensure utilization of entitlements, (3) develop modalities to extend coverage of maternity entitlements to the informal sector, (4) fully cover paid leave entitlements from social insurance or public fund sources, and (5) revisit limitations of paternity leave entitlements to those legally married. The Philippine policies on maternity protection operate separately from breastfeeding promotion programs, resulting in poor recognition of how maternity entitlements are intended to enable breastfeeding. Breastfeeding advocacy should be strengthened in workplaces through partnering with local government and health agencies for educational materials, advocacies with the employers and employees, and other modes of technical assistance. Strengthening and expanding the implementation of Mother-Baby Friendly Workplaces Certification will also improve the utilization of maternity entitlements in the workplaces on practicing breastfeeding.

### Electronic supplementary material

Below is the link to the electronic supplementary material.

**Additional file 1**: Data collection tools. Data collection tools used for the in-depth interviews of pregnant women, mothers of infants and their partners, employers, and key informants from the government and non-government organizations.

**Additional file 2**: Adoption of International Labour Organization’s Maternity Protection Convention and Recommendation in Philippine Policies. Provides a list of Philippine laws where ILO recommendations on maternity protection are reflected.

**Additional file 3**: Summary of provisions of Philippine policies related to maternity protection. Provides a description of policy provisions relating to maternity protection as reflected in Philippine laws.

**Additional file 4**: Key maternity protection provisions in the Philippines over time. Provides a timeline of the adoption of maternity protection policies in the Philippines.

## Data Availability

The datasets supporting the conclusions of this article are included within the article and its additional files.

## Abbreviations

CHD: Centers for Health Development
CSC: Civil Service Commission
DILG: Department of the Interior and Local Government
DOH: Department of Health
DOLE: Department of Labor and Employment
EMLL: 105-Day Expanded Maternity Leave Law
GNP: Gross National Product
IDI: In-Depth Interviews
ILO: International Labour Organization
IRR: Implementing Rules and Regulations
LGU: Local Government Units
NCP: Nutrition Center of the Philippines
PhilHealth: Philippine Health Insurance Corporation
PIN: Personal Identification Number
SSS: Social Security System
WHO: World Health Organization
