# Supplementary material for: Maternity protection policies and the enabling environment for breastfeeding in the Philippines: a qualitative study
Source: Int Breastfeed J. 2023 Nov 10;18:60. doi: 10.1186/s13006-023-00594-w (PMC10638739; doi:10.1186/s13006-023-00594-w)
Supplement: Supplementary file 2 — Additional file 2: Adoption of International Labour Organization’s Maternity Protection Convention and Recommendation in Philippine Policies. Provides a list of Philippine laws where ILO recommendations on maternity protection are reflected. [file 13006_2023_594_MOESM2_ESM.docx]

# **Additional file 2.** Adoption of the International Labour Organization’s Maternity Protection Convention and Recommendation in Philippine Policies

| International Labour Organization’s (ILO)  Maternity Protection Convention (Convention No.183) &  Maternity Protection Recommendation (Recommendation No. 191) | Adoption in Philippine Policies |
| --- | --- |
| Maternity Leave | |
| Shall be entitled to a period of maternity leave of not less than 14 weeks (Convention No. 183) | Republic Act (RA) 11210 or the 105-Day Expanded Maternity Leave Law (EMLL) (2019) |
| Woman is entitled to choose freely the time she takes her maternity leave, before or after childbirth (Recommendation No. 191) | Republic Act 8282 (1997), EMLL (2019) |
| Maternity Benefits | |
| Cash benefits to which woman is entitled during leave should be raised to full amount of woman’s previous earnings (Recommendation No. 191) | EMLL (2019) |
| Medical benefits shall be provided for the woman and her child in accordance with national laws and regulations (Convention No. 183) | Philippine Social Health Packages, Philippine Health Insurance Corporation (Philhealth) Circulars 022 -2014 and 025-2015, EMLL (2019) |
| Each Member shall ensure that the conditions to qualify for cash benefits can be satisfied by a large majority of the women to whom this Convention applies (Convention No. 183) | Presidential Decree (PD) 442 (1974), RA 8282 (1997), EMLL (2019) |
| Where a woman does not meet the conditions to qualify for cash benefits under national laws and regulations, she shall be entitled to adequate benefits out of social assistance funds (Convention No. 183) | EMLL (2019), Philhealth Circulars No. 022-2014, 025-2015 and 2017-0006 |
| In order to protect the situation of women in the labor market, benefits in respect of the leave shall be provided through compulsory social insurance or public funds, or in a manner determined by national law and practice. An employer shall not be individually liable for the direct cost of any such monetary benefit to a woman employed by him or her without that employer's specific agreement (Convention No. 183) | EMLL (2019) |
| Employment Protection and Non-Discrimination | |
| It shall be unlawful for an employer to terminate the employment of a woman during her pregnancy-related leave (Convention No. 183) | PD 442 (1974), EMLL (2019) |
| A woman is guaranteed the right to return to the same position or an equivalent position paid at the same rate at the end of her maternity leave (Convention No. 183) | PD 442 (1974), EMLL (2019) |
| Prohibition from requiring a test for pregnancy or a certificate of such a test when a woman is applying for employment, except on certain nature of work (Convention No. 183) | PD 442 (1974), EMLL (2019) |
| Health Protection | |
| Shall adopt appropriate measures to ensure that pregnant or breastfeeding women are not obliged to perform work assessed to establish a significant risk to the mother's health or that of her child (Convention No. 183) | PD 442 (1974), RA 9710 or Magna Carta of Women (2009) |
| Members should take measures to ensure assessment of any workplace risks related to the safety and health of the pregnant or nursing woman and her child (Recommendation No. 191) | PD 442 (1974), RA 9710 (2009) |
| A pregnant or nursing woman should not be obliged to do night work if a medical certificate declares such work to be incompatible with her pregnancy or nursing (Recommendation No. 191) | RA 10151 or the Act Allowing the Employment of Night Workers (2010) |
| A woman should be allowed to leave her workplace, if necessary, after notifying her employer, for the purpose of undergoing medical examinations relating to her pregnancy (Recommendation No. 191) | PD 442 (1974) |
| Breastfeeding | |
| A woman shall be provided with the right to one or more daily breaks or a daily reduction of hours of work to breastfeed her child | RA 10028 or the Expanded Breastfeeding Act of 2009 (2010) |
| The breaks or the reduction of daily hours of work shall be counted as working time and remunerated accordingly (Convention No. 183) | RA 10028 (2010) |
