# Supplementary material for: Maternity protection policies and the enabling environment for breastfeeding in the Philippines: a qualitative study
Source: Int Breastfeed J. 2023 Nov 10;18:60. doi: 10.1186/s13006-023-00594-w (PMC10638739; doi:10.1186/s13006-023-00594-w)
Supplement: Supplementary file 3 — Additional file 3: Summary of provisions of Philippine policies related to maternity protection. Provides a description of policy provisions relating to maternity protection as reflected in Philippine laws. [file 13006_2023_594_MOESM3_ESM.docx]

**Additional file 3.** Summary of provisions of Philippine policies related to maternity protection

| Philippine policy | Policy coverage | Description of main provisions |
| --- | --- | --- |
| Labor Code | | |
| Date Published:  1 May 1974  Presidential Decree (PD) No. 442  Labor Code of the Philippines | Maternity Leave  *Superseded by succeeding maternity leave policies including Republic Act (RA) 11210* | Granted pregnant woman employee who has rendered an aggregate service of at least 6 months for the last 12 months, maternity leave of at least 2 weeks prior to the expected date of delivery and another 4 weeks after normal delivery or abortion with full pay based on her regular or average weekly wages. Employers paid the benefits for the first four deliveries of the woman employee. |
|  | Additional leave for complication or illness | Provided extended maternity leave without pay on account of medically certified illness due to pregnancy, delivery, abortion, or miscarriage |
|  | Employment protection and non-discrimination | Prohibited: discrimination with respect to terms and conditions of employment solely on account of her sex; lesser compensation, salary, and benefits to female against a male employee; and favoring male employees with respect to promotion and other opportunities are acts of discrimination |
|  | Daycare facility | Required employers, in appropriate cases, to establish a nursery in the workplace for the benefit of women employees |
| Date Published:  21 June 2011  RA 10151  An Act Allowing the Employment of Night Workers thereby Repealing Articles 130 and 131 of PD 442, as amended, otherwise known as The Labor Code of the Philippines  *Amendment on PD No. 442* | Health protection | Provided protection to night workers who are certified as unfit for night work, due to health reasons such as periods of pregnancy, and time after birth, and additional periods necessary for the health of the mother and child; provisions shall not influence received maternity leave benefits  Tasked the Department of Labor and Employment (DOLE) to promulgate appropriate regulations in addition to existing ones to ensure protection, safety, and welfare of night workers |
|  | Enforcement and monitoring | Issued penalties for violations of the Act |
| Magna Carta of Women | | |
| Date Published:  14 August 2009  RA 9710  An Act Providing for the Magna Carta of Women | Additional leave for complication or illness | Provided a special benefit of 2 months leave with full pay to woman employees with service of at least six months for the last 12 months following surgery caused by gynecological disorders |
|  | Health protection | Provided of support services that will help enable women to balance their family obligations and work responsibilities including, but not limited to, the establishment of day care centers and breastfeeding rooms at the workplace as well as providing maternity leave pursuant to the Labor Code and other pertinent laws |
|  | Enforcement and monitoring | Mandated the Philippine Commission on Women (PCW) to: act as the overall monitoring body and oversight to ensure implementation of the act; establish incentives and awards systems to deserving entities and agencies that uphold rights of women and implement gender-responsive programs; monitor and develop indicators and guidelines for compliance to the Act (in coordination with other state agencies and the Commission on Human Rights (CHR)); and submit to Congress regular reports on the status and effectiveness of implementation of RA 9710  Tasked CHR to: assist in filing of cases against individuals, agencies, institutions that violate RA 9710; and recommend sanctions to the President or the Civil Service Commission (CSC) based on violations or non-compliance observed |
| Responsible Parenthood and Reproductive Health | | |
| Date Published:  21 December 2012  RA 10354  An Act Providing for a National Policy on Responsible Parenthood and Reproductive Health | Health protection | Provided comprehensive reproductive health care services and integration of responsible parenthood and family-planning programs: tasked local government units (LGU), with assistance from the Department of Health (DOH) to provide responsible parenthood and reproductive health care services and ensure that all public health facilities within the Service Delivery Network (SDN) provide modern family planning services to all clients; tasked DOH and LGUs to do public awareness and education campaigns and to provide training programs in coordination with other concerned agencies; provided appropriation for upgrading of facilities to meet Basic Emergency Maternal Obstetrics and Newborn Care/Comprehensive Emergency Obstetric and Newborn Care standards, training of health providers, and other requirement |
|  | Enforcement and monitoring | Defined the responsibilities of DOH and LGUs in: conducting regular monitoring of fund utilization for facility establishment or upgrading, developing specific guidelines for monitoring and evaluating effectiveness of SDNs |
| Social Security Benefits | | |
| Date Published:  18 June 1954  RA No.1161  ‘Social Security Law’, An Act to create a Social Security System providing Sickness, Unemployment, Retirement, Disability and Death Benefits for Employees | Maternity leave benefit | Provided compulsory Social Security System (SSS) coverage of all employees not over 60 years of age and their employers and of self-employed persons earning PHP 1800 or more per annum  Required that a covered female employee who has paid at least three monthly SSS contributions in the 12-month period preceding the semester of her childbirth shall be paid a daily maternity benefit equivalent to 100% of basic salary for 60 days |
| Date Published:  27 September 1977  PD 1202  Further amending RA 1161 Otherwise Known as Social Security Law | Maternity leave benefit | Provided that a female employee covered by SSS with at least three monthly maternity contributions in the 12 month period immediately preceding the semester of her childbirth shall be paid a daily salary credit for 45 days for the first four deliveries |
| Date Published:  25 July 1987  Executive Order No. 292 or the  Administrative Code of 1987 | Maternity leave benefit | Provided every woman in the government service who has rendered an aggregate of two or more years in service maternity leave of 60 days with full pay in addition to existing vacation and sick leave benefits |
| Date Published:  11 June 1996  RA 8187  Paternity Leave Act of 1996  *Superseded by RA 11210* | Paternity leave benefit | Granted paternity leave of seven days with full pay to all married employees in private and public sectors for the first four deliveries of the legitimate spouse with whom he is cohabiting and an optional additional 15 days paternity leave without pay |
|  | Enforcement and monitoring | Issued sanctions for any person, corporation, firm, or association who violates the law |
| Date Published:  22 July 1996  RA 8282  Social Security Act of 1997  *Amended RA 1161 for a strengthened and expanded coverage and increased benefits* | Maternity leave benefit | Granted a female SSS member who has paid at least three monthly contributions in a 12-month period immediately preceding the semester of childbirth or miscarriage and who was not able to work due to pregnancy, childbirth or miscarriage a maternity leave benefit equivalent to 100% of the average daily salary credit for 60 days for normal deliveries and 78 days for cesarean delivery for the first four deliveries or miscarriages |
|  | Enforcement and monitoring | Mandated SSS to keep records of operation/ transactions of funds, disbursements of accounts and to issue penal clause for violations |
| Date Published:  7 November 2000  RA 8972  Solo Parents’ Welfare Act of 2000 | Leave benefits for solo parents | Provided solo parents under the categories specified in the Act with comprehensive package of social development and welfare services and granted  solo parents with parental leave of not more than seven working days every year. Provided that employers shall allow flexible working schedule for solo parents unless exemption under certain ground was obtained from DOLE. |
| Date Published:  14 December 1998  CSC Memorandum Circular No. 41, series of 1998 -  Amendments to Rules 1 and XVI of the Omnibus Rules Implementing Book V of the Administrative Code of 1987 (EO 292) | Maternity leave benefit | Granted married women in government service who rendered an aggregate of two or more years of service a maternity leave of 60 calendar days with full pay. Those who have rendered less than two years are granted maternity leave with pay, amount of which is determined based on the length of service. Married contractual employees whether or not receiving 20% premium on their salary are entitled to maternity leave benefits like regular employees. |
| Date published:  23 October 2002  CSC Memorandum Circular No. 22, series of 2002 – Amendment of Section 11, Rule XVI of the Omnibus Rules Implementing Book V of the Administrative Code of 1987 | Maternity leave benefit | Granted all women in government service, married or unmarried, who rendered an aggregate of two or more years of service a maternity leave of 60 calendar days with full pay. Those who have rendered less than two years are granted maternity leave with pay, amount of which is determined based on the length of service. |
| Date Published:  7 February 2019  RA 11199  An Act Rationalizing and Expanding the Powers and Duties of the Social Security Commission to ensure the long -term viability of the Social Security System. Repealing for the Purpose Republic Act No. 1161. As amended by Republic Act No. 8282 otherwise known as the "Social Security Act of 1997". | Maternity leave benefit | Granted a female SSS member who has paid at least three monthly contributions in the 12-month period preceding the semester of childbirth a daily maternity benefit equivalent to 100% of her average daily salary credit for 60 days or 78 days in case of cesarean delivery for the first four deliveries or miscarriages. Self-employed women can avail of SSS benefits if she has covered contributions for both employer and employee. |
|  | Enforcement and monitoring | Issued penal clauses for violations under the provisions of the law and required SSS to submit an annual report of operations (including the number of persons covered and utilized SSS benefits and amount of benefits) to the President and Congress of the Philippines |
| Date Published:  20 February 2019  RA 11210  An Act increasing the Maternity Leave period to 105days of Female Workers with an option to extend for an Additional 30 days without pay, and Granting an additional 15 days for Solo Mothers, and for Other Purposes. Otherwise, known as the “105-Day Expanded Maternity Leave Law”  *Superseded RA 8282 and RA 8187* | Maternity leave benefit | Granted all female workers 105 days maternity leave with full pay, regardless if the delivery was normal or cesarean, and an option to extend for an additional 30 days without pay for every pregnancy regardless of frequency  Granted female workers who qualify under the 'Solo Parents Act' additional 15 days maternity leave with full pay  Granted 60 days maternity leave with full pay to female workers who suffered a miscarriage or emergency termination of pregnancy  For female workers in the public sector, full pay during maternity leave shall be paid by the agency where the female worker is employed. For the private sector, employers where tasked to pay the difference between the full salary and the cash benefit amount received from SSS |
|  | Employment protection and non-discrimination | Provided that utilization of benefits according to this law, from either the public or private sector, shall not be used as basis for demotion in employment or termination. Transfer to a parallel position or reassignment from one organizational unit to another agency is allowed, if it will not involve reduction in rank, status, salary or otherwise amount to constructive dismissal  Issued prohibitions on discrimination of employer against employment of women for the purpose of avoiding the provision of maternity benefits |
|  | Paternity leave | Allowed any female worker entitled to maternity benefits to allocate up to seven days of said benefits to the child's father, regardless of their civil status, and in the case of death or absence of a partner, to alternate caregiver within fourth degree of consanguinity or to a current partner sharing the same household |
|  | Maternity protection of non-employed women | SSS members classified as working in the informal economy and those who are voluntary contributors are also entitled to receive maternity benefits if they have remitted to SSS at least three monthly contributions in the 12 month period immediately preceding the semester of childbirth or emergency termination of pregnancy  Female workers who are neither voluntary nor regular members of SSS should be governed by Philippine Health Insurance Corporation (PhilHealth) Circular No. 022-2014 or “Social Health Insurance Coverage and Benefits for Women about to give Birth” |
|  | Enforcement and monitoring | Issued penalties/sanctions for those who fail to comply with the provisions of the law  Tasked the CSC, DOLE, SSS, and Gender Ombudsman of CHR, in consultation with trade unions, labor organizations, and employers' representatives to review maternity leave benefits of female workers in government service and private sector; required SSS and DOLE to submit valuation report every four years, and CSC every three years |
| Date Published:  1 May 2019  Implementing Rules and Regulations of RA No. 11210 | Maternity leave benefits | Provided the implementing rules and regulations of RA 11210 |
| Health Insurance | | |
| Date Published:  20 January 2017  Philhealth Circular No. 2017-0006 -  Strengthening the Implementation of the No Balance Billing Policy | Maternity health benefits/  health protection | Covered indigent members of the population, as identified by the Department of Social Welfare and Development (DSWD), under the No Balance Billing Policy to ensure that fees or expenses beyond PhilHealth packages will not be charged to the patient |
| Date Published:  14 December 2018  Philhealth Circular No. 2018-0021  Enhancement of Philhealth Newborn Package | Maternity health benefits/  health protection | Added the expanded newborn screening services to the covered essential health services for the newborns |
| Date Published:  14 October 2014  Philhealth Circular No. 022-2014  Social Health Insurance Coverage and Benefits for Women about to Give Birth  15 June 2015  PhilHealth Circular No. 025-2015 - Social Health Insurance Coverage and Benefits for Women about to Give Birth Revision 1 | Maternity health benefits/  health protection | Provided mechanisms to ensure social health insurance coverage for women who are about to give birth |
| Expanded Promotion of Breastfeeding | | |
| Date Published:  16 March 2010  RA 10028  An Act Expanding the Promotion of Breastfeeding, Amending for the Purpose RA No. 7600, otherwise known as "An Act Providing Incentives to all Government and Private Health Institutions with Rooming-In and Breastfeeding Practices and for other Purposes" Otherwise known as “Expanded Breastfeeding Promotion Act of 2009” | Breastfeeding promotion | Encouraged employers to avail of or develop, as part of human resource development services, breastfeeding and lactation support programs for working mothers, through coordination with the DOH and other relevant sectors |
|  | Lactation break for breastfeeding mothers | Granted nursing employees break intervals of not less than 40 minutes for every eight-hour working period, in addition to regular break time for meals |
|  | Workplace lactation facility | Mandated the establishment of lactation rooms in workplaces and provided guidelines or standards for the establishment of lactation rooms |
|  | Enforcement and monitoring | Issued sanctions for all private non-health facilities, establishments, and institutions which refuse or fail to comply with the establishment of lactation rooms and provision of lactation breaks |
| Date Published:  11 April 2011  Memorandum Circular 2011-54  Implementation and Monitoring of the National Policy on Breastfeeding and Setting-up of Workplace Lactation Program | Workplace lactation facility | Enjoined regional and local governments to adopt workplace lactation programs and encouraged the inclusion of the provision of lactation facility as part of private establishments’ business permit requirements |
| Date Published:  22 August 2011  Implementing Rules and Regulations of RA 10028 | Workplace lactation facility | Provided detailed guidelines on the implementation, monitoring and enforcement of RA 10028 |
| Date Published:  17 August 2015  CSC Memorandum Circular No. 12, s. 2015  Exemption from the Establishment of Lactation Station in Government Agencies | Workplace lactation facility | Provided the guidelines for granting exemptions to government agencies from establishing lactation stations, which include considerations for limited office space and the low percentage (10% or less) of female employees who are in the reproductive age. |
| Date Published:  20 May 2015  DOLE Department Order No. 143 s. 2015  Guidelines Governing Exemption of Establishments from Setting Up Workplace Lactations | Workplace lactation facility | Provided guidelines in granting exemptions from establishment of lactation facilities in private sector Criteria for exemption include the following: no lactating employee, no pregnant employee, no female clients who visit or transact with the establishment |
