# Supplementary material for: Maternity protection policies and the enabling environment for breastfeeding in the Philippines: a qualitative study
Source: Int Breastfeed J. 2023 Nov 10;18:60. doi: 10.1186/s13006-023-00594-w (PMC10638739; doi:10.1186/s13006-023-00594-w)
Supplement: Supplementary file 4 — Additional file 4: Key maternity protection provisions in the Philippines over time. Provides a timeline of the adoption of maternity protection policies in the Philippines. [file 13006_2023_594_MOESM4_ESM.docx]

**Additional file 4.** Key maternity protection provisions in the Philippines over time

| Key rights of working women | 1974-1995 | 1996- 2002 | 2003-2008 | 2009-2018 | 2019 - present |
| --- | --- | --- | --- | --- | --- |
| Equity at work | ✓ | ✓ | ✓ | ✓ | ✓ |
| Health protection | | | | | |
| Appropriate measures to ensure safe work set-up for the pregnant mother | ✓ | ✓ | ✓ | ✓ | ✓ |
| Not obliged for night work |  |  | ✓ | ✓ | ✓ |
| Leave related to medical conditions during pregnancy | ✓ | ✓ | ✓ | ✓ | ✓ |
| Maternity leave and entitlements | | | | | |
| Duration of maternity leave | 45 days for private sector  60 days for public sector | 60 days for vaginal delivery/78 days for caesarean delivery for private sector  60 days for public sector | 60 days for vaginal delivery/78 days for caesarean delivery for private sector  60 days for public sector | 60 days for vaginal delivery/78 days for caesarean delivery for private sector  60 days for public sector | 105 days (with pay) and optional additional 30 days (without pay) |
| Leave for solo parent |  | up to 7 days every year | up to 7 days every year | up to 7 days every year | up to 7 days per year plus additional 15 days (fully-paid) during the maternity period |
| Amount of cash benefits | 100% of daily salary credit from SSS for private sector  100% of salary for public sector | 100% of daily salary credit from SSS for private sector  100% of salary for public sector | 100% of daily salary credit from SSS for private sector  100% of salary for public sector | 100% of daily salary credit from SSS for private sector  100% of salary for public sector | 100% of salary for 105 days for both private and public sector |
| Coverage of cash benefits | private sector employees who satisfy conditions on paid contributions to SSS  married workers in the public sector who satisfy conditions on employment status and duration | private sector employees who satisfy conditions on SSS contributions payment  married workers in the public sector who satisfy conditions on employment status and duration | private sector employees who satisfy conditions on SSS contributions payment  all workers in the public sector who have rendered at least two years of service; those in service for less than 2 years - entitled to receive a portion of their salary depending on the length of service | private sector employees who satisfy conditions on SSS contributions payment  all workers in the public sector who have rendered at least two years of service; those in service for less than 2 years - entitled to receive a portion of their salary depending on the length of service | private sector employees who satisfy conditions on SSS contributions payment  all workers in the public sector |
| Monetary source of cash benefits | employer | social security fund for private sector  agency budget for public sector | social security fund for private sector  agency budget for public sector | social security fund for private sector  agency budget for public sector | mixed (social security and employers) for private sector  agency budget for public sector |
| Lactation support (workplace) | | | | | |
| Paid lactation break |  |  |  | ✓ | ✓ |
| Lactation spaces |  |  |  | ✓ | ✓ |
| Maternity protection for the informal sector workers | | | | | |
| Maternity cash provisions |  | those who satisfy conditions on paid contributions to SSS | those who satisfy conditions on paid contributions to SSS | those who satisfy conditions on paid contributions to SSS | those who satisfy conditions on paid contributions to SSS |
| Others; social assistance |  |  | ✓ | ✓ | ✓ |
| Paternity leave | | | | | |
| Duration |  | 7 days (with pay) and additional 15 days (w/o pay) | 7 days (with pay) and additional 15 days (w/o pay) | 7 days (with pay) and additional 15 days (w/o pay) | 7 days (with pay) and additional 15 days (w/o pay)  maternity leave credits of up to 7 days that the mother can transfer to the father of the child or an alternate caregiver |
| Coverage |  | for legitimate spouse of the mother | for legitimate spouse of the mother | for legitimate spouse of the mother | for the legitimate spouse of the mother (for the seven days leave)  transferrable leave applies for the father or any  caregiver within fourth degree of consanguinity or to a current partner sharing the same household with the mother |
